# 5-Bromo-2,4,6-trimethyl-3-(3-methyl­phenyl­sulfin­yl)-1-benzo­furan

**DOI:** 10.1107/S1600536814005352

**Published:** 2014-03-15

**Authors:** Hong Dae Choi, Pil Ja Seo, Uk Lee

**Affiliations:** a Department of Chemistry, Dongeui University, San 24 Kaya-dong, Busanjin-gu, Busan 614-714, Republic of Korea; b Department of Chemistry, Pukyong National University, 599-1 Daeyeon 3-dong, Nam-gu, Busan 608-737, Republic of Korea

## Abstract

In the title compound, C_18_H_17_BrO_2_S, the dihedral angle between the mean plane of the benzo­furan ring system and the benzene ring is 68.58 (4)°. In the crystal, mol­ecules are linked *via* pairs of C—H⋯O hydrogen bonds into inversion dimers. These dimers are linked by C—H⋯O hydrogen bonds and π–π inter­actions between the benzene rings of neighbouring mol­ecules [centroid–centroid distance = 3.783 (1) Å], forming a three-dimensional network. In addition, the stacked mol­ecules exhibit inversion-related S⋯O contacts [3.153 (1) Å] involving the sulfinyl groups.

## Related literature   

For background information and the crystal structures of related compounds, see: Choi *et al.* (2008 ▶, 2011 ▶). For details of sulfin­yl–sulfinyl inter­actions, see: Choi *et al.* (2013 ▶) and for a review of carbon­yl–carbonyl inter­actions, see: Allen *et al.* (1998 ▶).
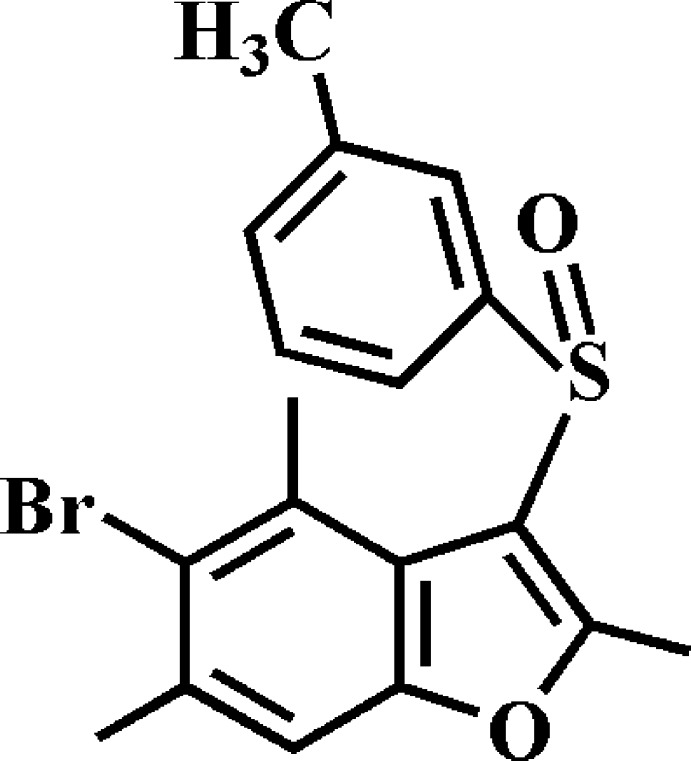


## Experimental   

### 

#### Crystal data  

C_18_H_17_BrO_2_S
*M* *_r_* = 377.29
Triclinic, 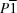

*a* = 6.2336 (1) Å
*b* = 11.0353 (2) Å
*c* = 12.9149 (2) Å
α = 69.384 (1)°
β = 76.421 (1)°
γ = 76.799 (1)°
*V* = 797.84 (2) Å^3^
*Z* = 2
Mo *K*α radiation
μ = 2.71 mm^−1^
*T* = 173 K
0.35 × 0.34 × 0.28 mm

#### Data collection  

Bruker SMART APEXII CCD diffractometer
Absorption correction: multi-scan (*SADABS*; Bruker, 2009 ▶) *T*_min_ = 0.462, *T*_max_ = 0.746
14962 measured reflections
4023 independent reflections
3702 reflections with *I* > 2σ(*I*)
*R*_int_ = 0.031

#### Refinement  

*R*[*F* ^2^ > 2σ(*F* ^2^)] = 0.026
*wR*(*F* ^2^) = 0.070
*S* = 1.06
4023 reflections
203 parameters
H-atom parameters constrained
Δρ_max_ = 0.36 e Å^−3^
Δρ_min_ = −0.49 e Å^−3^

### 

Data collection: *APEX2* (Bruker, 2009 ▶); cell refinement: *SAINT* (Bruker, 2009 ▶); data reduction: *SAINT*; program(s) used to solve structure: *SHELXS97* (Sheldrick, 2008 ▶); program(s) used to refine structure: *SHELXL97* (Sheldrick, 2008 ▶); molecular graphics: *ORTEP-3 for Windows* (Farrugia, 2012 ▶) and *DIAMOND* (Brandenburg, 1998 ▶); software used to prepare material for publication: *SHELXL97*.

## Supplementary Material

Crystal structure: contains datablock(s) I. DOI: 10.1107/S1600536814005352/bx2455sup1.cif

Structure factors: contains datablock(s) I. DOI: 10.1107/S1600536814005352/bx2455Isup2.hkl

Supporting information file. DOI: 10.1107/S1600536814005352/bx2455Isup3.cml

CCDC reference: 990682

Additional supporting information:  crystallographic information; 3D view; checkCIF report

## Figures and Tables

**Table 1:** Hydrogen-bond geometry (Å, °)

*D*—H⋯*A*	*D*—H	H⋯*A*	*D*⋯*A*	*D*—H⋯*A*
C6—H6⋯O1^i^	0.95	2.50	3.4478 (19)	172
C11—H11*A*⋯O2^ii^	0.98	2.36	3.244 (2)	150

Symmetry codes: (i) 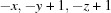; (ii) 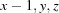.

**Fig. 1.**
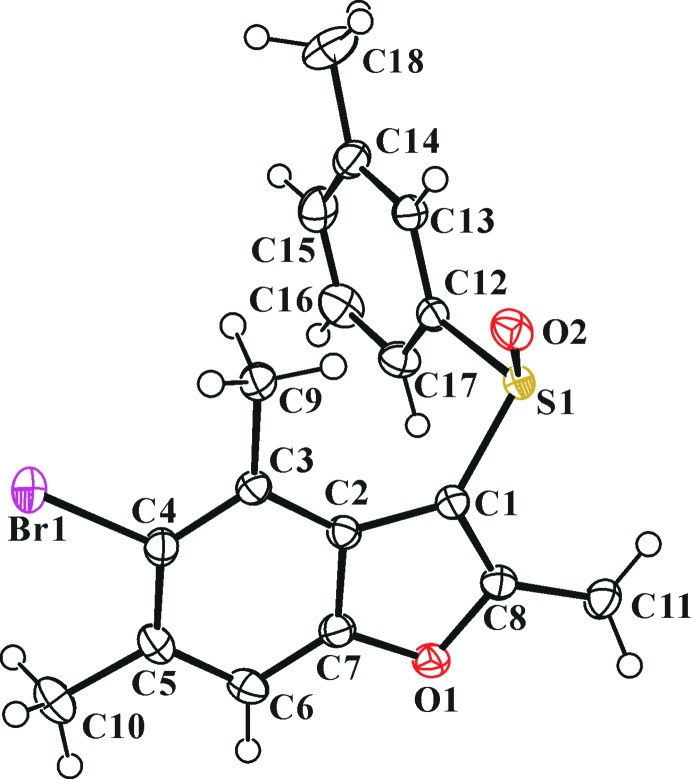
The molecular structure of the title molecule with the atom numbering scheme The displacement ellipsoids are drawn at the 50% probability level. The hydrogen atoms are presented as small spheres of arbitrary radius.

**Fig. 2.**
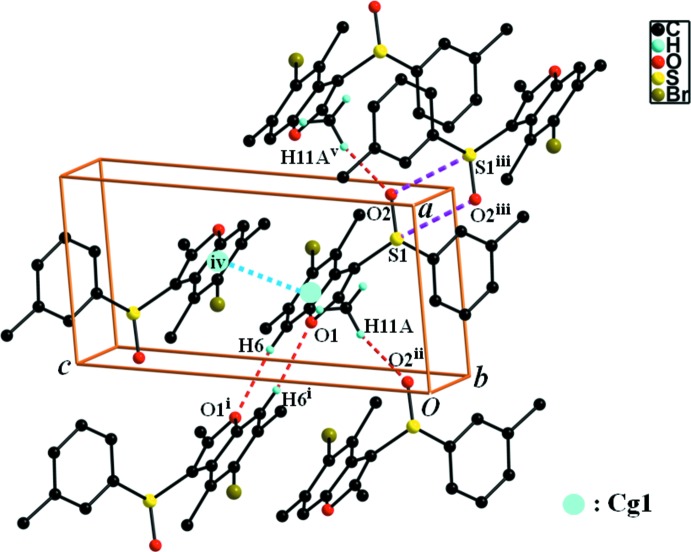
A view of the C—H···O, π···π and S···O interactions (dotted lines) in the crystal structure of the title compound. H atoms non-participating in hydrogen-bonding were omitted for clarity. [Symmetry codes: (i) - x, - y + 1, - z + 1; (ii) x - 1, y, z; (iii) - x + 2, - y + 1, - z; (iv) - x + 1, -y + 1, - z + 1; (v) x + 1, y, z.]

## supplementary crystallographic information

### 1. Comment

As a part of our continuing study of 5-bromo-2,4,6-trimethyl-1-benzofuran derivatives containing phenylsulfinyl (Choi *et al.*, 2008) and 4-fluorophenylsulfinyl (Choi *et al.*, 2011) substituents in the 3-position, we report herein on the crystal structure of the title compound.

In the title molecule (Fig. 1), the benzofuran unit is essentially planar, with a mean deviation of 0.027 (1) Å from the least-squares plane defined by the nine constituent atoms. The 3-methylphenyl ring is essentially planar, with a mean deviation of 0.011 (1) Å from the least-squares plane defined by the six constituent atoms. The dihedral angle formed by the benzofuran ring system and the 3-methylphenyl ring is 68.58 (4)°.

In the crystal structure (Fig. 2), molecules are linked via pairs of C–H···O hydrogen bonds into inversion dimers (Table 1). These dimers are further packed by C—H···O hydrogen bonds (Table 1) and π···π interactions between the benzene rings of neighbouring molecules, with a Cg1···Cg1^iv^ distance of 3.783 (1) Å and an interplanar distance of 3.402 (1) Å resulting in a slippage of 1.655 (1) Å (Cg1 is the centroid of the C2–C7 benzene ring), forming a three-dimensional network. In addition, the crystal packing (Fig. 2) exhibits a sulfinyl–sulfinyl interaction (Choi *et al.*, 2013) interpreted as similar to a type II carbonyl–carbonyl interaction (Allen *et al.* 1998), with S1···O2^iii^ and O2^iii^···S1 distance of 3.153 (1) Å.

### 2. Experimental

3-Chloroperoxybenzoic acid (77%, 269 mg, 1.2 mmol) was added in small portions to a stirred solution of 5-bromo-2,4,6-trimethyl-3-(3-methylphenylsulfanyl)-1-benzofuran (397 mg, 1.1 mmol) in dichloromethane (35 mL) at 273 K. After being stirred at room temperature for 5h, the mixture was washed with saturated sodium bicarbonate solution and the organic layer was separated, dried over magnesium sulfate, filtered and concentrated at reduced pressure. The residue was purified by column chromatography (hexane–ethyl acetate, 2:1 *v*/*v*) to afford the title compound as a colorless solid [yield 72%, m.p. 469–470 K; *R*_f_ = 0.52 (hexane–ethyl acetate, 2:1 *v*/*v*)]. Single crystals suitable for X-ray diffraction were prepared by slow evaporation of a solution of the title compound in ethyl acetate at room temperature.

### 3. Refinement

All H atoms were positioned geometrically and refined using a riding model, with C—H = 0.95 Å for aryl and 0.95 Å for methyl H atoms. *U*_iso_ (H) = 1.2*U*_eq_ (C) for aryl and 1.5*U*_eq_ (C) for methyl H atoms. The positions of methyl hydrogens were optimized using the *SHELXL*-97's command AFIX 137 (Sheldrick, 2008).

### Crystal data

**Table:**

C_18_H_17_BrO_2_S	*Z* = 2
*M**_r_* = 377.29	*F*(000) = 384
Triclinic, *P*1	*D*_x_ = 1.570 Mg m^−^^3^
Hall symbol: -P 1	Melting point = 469–470 K
*a* = 6.2336 (1) Å	Mo *K*α radiation, λ = 0.71073 Å
*b* = 11.0353 (2) Å	Cell parameters from 8866 reflections
*c* = 12.9149 (2) Å	θ = 3.0–28.4°
α = 69.384 (1)°	µ = 2.71 mm^−^^1^
β = 76.421 (1)°	*T* = 173 K
γ = 76.799 (1)°	Block, colourless
*V* = 797.84 (2) Å^3^	0.35 × 0.34 × 0.28 mm

### Data collection

**Table:**

Bruker SMART APEXII CCD diffractometer	4023 independent reflections
Radiation source: rotating anode	3702 reflections with *I* > 2σ(*I*)
Graphite multilayer monochromator	*R*_int_ = 0.031
Detector resolution: 10.0 pixels mm^-1^	θ_max_ = 28.4°, θ_min_ = 1.7°
φ and ω scans	*h* = −8→8
Absorption correction: multi-scan (*SADABS*; Bruker, 2009)	*k* = −14→14
*T*_min_ = 0.462, *T*_max_ = 0.746	*l* = −17→17
14962 measured reflections	

### Refinement

**Table:**

Refinement on *F*^2^	Primary atom site location: structure-invariant direct methods
Least-squares matrix: full	Secondary atom site location: difference Fourier map
*R*[*F*^2^ > 2σ(*F*^2^)] = 0.026	Hydrogen site location: difference Fourier map
*wR*(*F*^2^) = 0.070	H-atom parameters constrained
*S* = 1.06	*w* = 1/[σ^2^(*F*_o_^2^) + (0.0354*P*)^2^ + 0.3061*P*] where *P* = (*F*_o_^2^ + 2*F*_c_^2^)/3
4023 reflections	(Δ/σ)_max_ = 0.001
203 parameters	Δρ_max_ = 0.36 e Å^−^^3^
0 restraints	Δρ_min_ = −0.49 e Å^−^^3^

### Special details

**Table:**

Geometry. All esds (except the esd in the dihedral angle between two l.s. planes) are estimated using the full covariance matrix. The cell esds are taken into account individually in the estimation of esds in distances, angles and torsion angles; correlations between esds in cell parameters are only used when they are defined by crystal symmetry. An approximate (isotropic) treatment of cell esds is used for estimating esds involving l.s. planes.
Refinement. Refinement of F^2^ against ALL reflections. The weighted R-factor wR and goodness of fit S are based on F^2^, conventional R-factors R are based on F, with F set to zero for negative F^2^. The threshold expression of F^2^ > 2sigma(F^2^) is used only for calculating R-factors(gt) etc. and is not relevant to the choice of reflections for refinement. R-factors based on F^2^ are statistically about twice as large as those based on F, and R- factors based on ALL data will be even larger.

### Fractional atomic coordinates and isotropic or equivalent isotropic displacement parameters (Å^2^)

**Table:**

	*x*	*y*	*z*	*U*_iso_*/*U*_eq_	
Br1	0.62873 (3)	0.900826 (16)	0.403993 (14)	0.03223 (7)	
S1	0.75692 (6)	0.51077 (3)	0.11568 (3)	0.01987 (9)	
O1	0.27755 (19)	0.45708 (11)	0.37436 (9)	0.0241 (2)	
O2	0.99335 (19)	0.51170 (11)	0.12084 (10)	0.0265 (2)	
C1	0.5810 (3)	0.51004 (14)	0.24439 (12)	0.0196 (3)	
C2	0.5305 (2)	0.59855 (14)	0.31119 (12)	0.0190 (3)	
C3	0.6265 (3)	0.69926 (14)	0.31564 (12)	0.0195 (3)	
C4	0.5100 (3)	0.76127 (15)	0.39418 (13)	0.0219 (3)	
C5	0.3111 (3)	0.72874 (16)	0.46735 (13)	0.0244 (3)	
C6	0.2268 (3)	0.62354 (17)	0.46533 (13)	0.0252 (3)	
H6	0.0964	0.5954	0.5154	0.030*	
C7	0.3399 (3)	0.56176 (15)	0.38791 (12)	0.0214 (3)	
C8	0.4262 (3)	0.42809 (15)	0.28649 (12)	0.0221 (3)	
C9	0.8457 (3)	0.73299 (16)	0.24263 (14)	0.0242 (3)	
H9A	0.8171	0.8110	0.1782	0.036*	
H9B	0.9253	0.6592	0.2159	0.036*	
H9C	0.9372	0.7506	0.2863	0.036*	
C10	0.1860 (3)	0.80420 (19)	0.54665 (16)	0.0341 (4)	
H10A	0.0587	0.7619	0.5938	0.051*	
H10B	0.1318	0.8943	0.5033	0.051*	
H10C	0.2863	0.8054	0.5943	0.051*	
C11	0.3854 (3)	0.31944 (16)	0.25548 (14)	0.0270 (3)	
H11A	0.2470	0.3462	0.2240	0.041*	
H11B	0.3713	0.2422	0.3224	0.041*	
H11C	0.5108	0.2979	0.1995	0.041*	
C12	0.6482 (3)	0.67448 (14)	0.03546 (12)	0.0198 (3)	
C13	0.7999 (3)	0.75067 (15)	−0.04158 (12)	0.0225 (3)	
H13	0.9557	0.7183	−0.0474	0.027*	
C14	0.7230 (3)	0.87472 (16)	−0.11039 (14)	0.0275 (3)	
C15	0.4952 (3)	0.92001 (17)	−0.09772 (15)	0.0329 (4)	
H15	0.4411	1.0057	−0.1425	0.039*	
C16	0.3449 (3)	0.84324 (19)	−0.02136 (15)	0.0337 (4)	
H16	0.1893	0.8765	−0.0144	0.040*	
C17	0.4195 (3)	0.71802 (17)	0.04509 (14)	0.0272 (3)	
H17	0.3168	0.6635	0.0959	0.033*	
C18	0.8835 (4)	0.9564 (2)	−0.19860 (17)	0.0443 (5)	
H18A	0.8480	0.9733	−0.2729	0.066*	
H18B	1.0366	0.9092	−0.1953	0.066*	
H18C	0.8703	1.0399	−0.1850	0.066*	

### Atomic displacement parameters (Å^2^)

**Table:**

	*U*^11^	*U*^22^	*U*^33^	*U*^12^	*U*^13^	*U*^23^
Br1	0.04364 (12)	0.02725 (10)	0.03175 (10)	−0.01115 (8)	−0.00252 (8)	−0.01557 (7)
S1	0.02068 (18)	0.01787 (17)	0.02010 (17)	−0.00149 (13)	−0.00020 (13)	−0.00790 (13)
O1	0.0248 (6)	0.0267 (6)	0.0223 (5)	−0.0103 (5)	0.0007 (4)	−0.0087 (4)
O2	0.0194 (5)	0.0287 (6)	0.0292 (6)	0.0009 (4)	−0.0019 (4)	−0.0109 (5)
C1	0.0212 (7)	0.0183 (6)	0.0185 (6)	−0.0026 (5)	−0.0023 (5)	−0.0058 (5)
C2	0.0199 (7)	0.0189 (7)	0.0174 (6)	−0.0019 (5)	−0.0029 (5)	−0.0054 (5)
C3	0.0205 (7)	0.0181 (6)	0.0187 (6)	−0.0028 (5)	−0.0033 (5)	−0.0047 (5)
C4	0.0260 (8)	0.0195 (7)	0.0216 (7)	−0.0031 (6)	−0.0054 (6)	−0.0076 (6)
C5	0.0247 (8)	0.0268 (8)	0.0220 (7)	0.0008 (6)	−0.0034 (6)	−0.0113 (6)
C6	0.0215 (7)	0.0319 (8)	0.0213 (7)	−0.0058 (6)	0.0009 (6)	−0.0093 (6)
C7	0.0226 (7)	0.0215 (7)	0.0199 (7)	−0.0058 (6)	−0.0030 (6)	−0.0054 (5)
C8	0.0253 (8)	0.0212 (7)	0.0190 (7)	−0.0041 (6)	−0.0039 (6)	−0.0052 (5)
C9	0.0231 (7)	0.0237 (7)	0.0269 (7)	−0.0071 (6)	0.0008 (6)	−0.0106 (6)
C10	0.0325 (9)	0.0390 (10)	0.0318 (9)	0.0002 (8)	0.0010 (7)	−0.0204 (8)
C11	0.0324 (9)	0.0236 (8)	0.0282 (8)	−0.0094 (7)	−0.0055 (7)	−0.0085 (6)
C12	0.0223 (7)	0.0191 (7)	0.0180 (6)	−0.0024 (6)	−0.0027 (5)	−0.0069 (5)
C13	0.0242 (8)	0.0235 (7)	0.0213 (7)	−0.0064 (6)	−0.0022 (6)	−0.0084 (6)
C14	0.0363 (9)	0.0243 (8)	0.0239 (7)	−0.0107 (7)	−0.0056 (7)	−0.0061 (6)
C15	0.0436 (11)	0.0230 (8)	0.0299 (8)	0.0011 (7)	−0.0140 (8)	−0.0047 (6)
C16	0.0266 (9)	0.0370 (10)	0.0324 (9)	0.0061 (7)	−0.0082 (7)	−0.0097 (7)
C17	0.0226 (8)	0.0304 (8)	0.0253 (8)	−0.0026 (6)	−0.0018 (6)	−0.0071 (6)
C18	0.0531 (13)	0.0348 (10)	0.0388 (10)	−0.0213 (9)	−0.0083 (9)	0.0051 (8)

### Geometric parameters (Å, º)

**Table:**

Br1—C4	1.9073 (15)	C9—H9C	0.9800
S1—O2	1.4934 (12)	C10—H10A	0.9800
S1—C1	1.7619 (15)	C10—H10B	0.9800
S1—C12	1.8024 (15)	C10—H10C	0.9800
S1—O2^i^	3.1527 (12)	C11—H11A	0.9800
O1—C8	1.3694 (19)	C11—H11B	0.9800
O1—C7	1.3770 (18)	C11—H11C	0.9800
C1—C8	1.361 (2)	C12—C13	1.387 (2)
C1—C2	1.457 (2)	C12—C17	1.387 (2)
C2—C7	1.393 (2)	C13—C14	1.392 (2)
C2—C3	1.401 (2)	C13—H13	0.9500
C3—C4	1.393 (2)	C14—C15	1.385 (3)
C3—C9	1.508 (2)	C14—C18	1.505 (2)
C4—C5	1.405 (2)	C15—C16	1.381 (3)
C5—C6	1.390 (2)	C15—H15	0.9500
C5—C10	1.510 (2)	C16—C17	1.386 (2)
C6—C7	1.375 (2)	C16—H16	0.9500
C6—H6	0.9500	C17—H17	0.9500
C8—C11	1.479 (2)	C18—H18A	0.9800
C9—H9A	0.9800	C18—H18B	0.9800
C9—H9B	0.9800	C18—H18C	0.9800
			
O2—S1—C1	111.08 (7)	C5—C10—H10A	109.5
O2—S1—C12	106.70 (7)	C5—C10—H10B	109.5
C1—S1—C12	96.91 (7)	H10A—C10—H10B	109.5
O2—S1—O2^i^	78.25 (6)	C5—C10—H10C	109.5
C1—S1—O2^i^	169.73 (6)	H10A—C10—H10C	109.5
C12—S1—O2^i^	83.97 (5)	H10B—C10—H10C	109.5
C8—O1—C7	106.60 (12)	C8—C11—H11A	109.5
C8—C1—C2	107.12 (13)	C8—C11—H11B	109.5
C8—C1—S1	118.68 (12)	H11A—C11—H11B	109.5
C2—C1—S1	133.04 (11)	C8—C11—H11C	109.5
C7—C2—C3	119.45 (14)	H11A—C11—H11C	109.5
C7—C2—C1	104.27 (13)	H11B—C11—H11C	109.5
C3—C2—C1	136.25 (14)	C13—C12—C17	121.63 (14)
C4—C3—C2	115.39 (14)	C13—C12—S1	117.57 (12)
C4—C3—C9	123.20 (13)	C17—C12—S1	120.62 (12)
C2—C3—C9	121.37 (13)	C12—C13—C14	119.71 (15)
C3—C4—C5	125.00 (14)	C12—C13—H13	120.1
C3—C4—Br1	117.82 (11)	C14—C13—H13	120.1
C5—C4—Br1	117.18 (11)	C15—C14—C13	118.57 (16)
C6—C5—C4	118.23 (14)	C15—C14—C18	120.73 (16)
C6—C5—C10	119.23 (15)	C13—C14—C18	120.69 (17)
C4—C5—C10	122.54 (15)	C16—C15—C14	121.38 (16)
C7—C6—C5	117.29 (14)	C16—C15—H15	119.3
C7—C6—H6	121.4	C14—C15—H15	119.3
C5—C6—H6	121.4	C15—C16—C17	120.44 (17)
C6—C7—O1	124.56 (14)	C15—C16—H16	119.8
C6—C7—C2	124.45 (14)	C17—C16—H16	119.8
O1—C7—C2	110.98 (13)	C16—C17—C12	118.19 (16)
C1—C8—O1	110.99 (13)	C16—C17—H17	120.9
C1—C8—C11	133.49 (15)	C12—C17—H17	120.9
O1—C8—C11	115.50 (14)	C14—C18—H18A	109.5
C3—C9—H9A	109.5	C14—C18—H18B	109.5
C3—C9—H9B	109.5	H18A—C18—H18B	109.5
H9A—C9—H9B	109.5	C14—C18—H18C	109.5
C3—C9—H9C	109.5	H18A—C18—H18C	109.5
H9A—C9—H9C	109.5	H18B—C18—H18C	109.5
H9B—C9—H9C	109.5		
			
O2—S1—C1—C8	−136.06 (12)	C8—O1—C7—C2	−1.35 (16)
C12—S1—C1—C8	113.02 (13)	C3—C2—C7—C6	4.5 (2)
O2—S1—C1—C2	58.06 (17)	C1—C2—C7—C6	−177.07 (15)
C12—S1—C1—C2	−52.85 (16)	C3—C2—C7—O1	−176.74 (13)
C8—C1—C2—C7	−1.46 (16)	C1—C2—C7—O1	1.73 (16)
S1—C1—C2—C7	165.60 (13)	C2—C1—C8—O1	0.71 (17)
C8—C1—C2—C3	176.62 (17)	S1—C1—C8—O1	−168.54 (10)
S1—C1—C2—C3	−16.3 (3)	C2—C1—C8—C11	179.18 (16)
C7—C2—C3—C4	−4.3 (2)	S1—C1—C8—C11	9.9 (2)
C1—C2—C3—C4	177.88 (16)	C7—O1—C8—C1	0.35 (17)
C7—C2—C3—C9	173.53 (14)	C7—O1—C8—C11	−178.42 (13)
C1—C2—C3—C9	−4.3 (3)	O2—S1—C12—C13	25.77 (14)
C2—C3—C4—C5	0.9 (2)	C1—S1—C12—C13	140.27 (12)
C9—C3—C4—C5	−176.88 (15)	O2—S1—C12—C17	−158.95 (13)
C2—C3—C4—Br1	−179.31 (10)	C1—S1—C12—C17	−44.45 (14)
C9—C3—C4—Br1	2.9 (2)	C17—C12—C13—C14	0.8 (2)
C3—C4—C5—C6	2.7 (2)	S1—C12—C13—C14	176.07 (12)
Br1—C4—C5—C6	−177.13 (12)	C12—C13—C14—C15	1.6 (2)
C3—C4—C5—C10	−176.77 (15)	C12—C13—C14—C18	−176.88 (16)
Br1—C4—C5—C10	3.4 (2)	C13—C14—C15—C16	−2.1 (3)
C4—C5—C6—C7	−2.7 (2)	C18—C14—C15—C16	176.39 (18)
C10—C5—C6—C7	176.80 (15)	C14—C15—C16—C17	0.1 (3)
C5—C6—C7—O1	−179.40 (14)	C15—C16—C17—C12	2.3 (3)
C5—C6—C7—C2	−0.8 (2)	C13—C12—C17—C16	−2.8 (2)
C8—O1—C7—C6	177.44 (15)	S1—C12—C17—C16	−177.85 (13)

Symmetry code: (i) −*x*+2, −*y*+1, −*z*.

### Hydrogen-bond geometry (Å, º)

**Table:**

*D*—H···*A*	*D*—H	H···*A*	*D*···*A*	*D*—H···*A*
C6—H6···O1^ii^	0.95	2.50	3.4478 (19)	172
C11—H11*A*···O2^iii^	0.98	2.36	3.244 (2)	150

Symmetry codes: (ii) −*x*, −*y*+1, −*z*+1; (iii) *x*−1, *y*, *z*.

## References

[bb1] Allen, F. H., Baalham, C. A., Lommerse, J. P. M. & Raithby, P. R. (1998). *Acta Cryst.* B**54**, 320–329.

[bb2] Brandenburg, K. (1998). *DIAMOND* Crystal Impact GbR, Bonn, Germany.

[bb3] Bruker (2009). *APEX2*, *SADABS* and *SAINT* Bruker AXS Inc., Madison, Wisconsin, USA.

[bb4] Choi, H. D., Seo, P. J. & Lee, U. (2013). *Acta Cryst.* E**69**, o820. 10.1107/S1600536813011793 PMC3684910 23795012

[bb5] Choi, H. D., Seo, P. J., Son, B. W. & Lee, U. (2008). *Acta Cryst.* E**64**, o1826. 10.1107/S160053680802669X PMC2960548 21201801

[bb6] Choi, H. D., Seo, P. J., Son, B. W. & Lee, U. (2011). *Acta Cryst.* E**67**, o471. 10.1107/S1600536811002303 PMC3051696 21523130

[bb7] Farrugia, L. J. (2012). *J. Appl. Cryst.* **45**, 849–854.

[bb8] Sheldrick, G. M. (2008). *Acta Cryst.* A**64**, 112–122. 10.1107/S0108767307043930 18156677

